# Relationship between fiducial points on the peripheral and central blood pressure waveforms: rate of rise of the central waveform is a determinant of peripheral systolic blood pressure

**DOI:** 10.1152/ajpheart.00818.2020

**Published:** 2021-02-19

**Authors:** Ye Li, Antoine Guilcher, Peter H. Charlton, Samuel Vennin, Jordi Alastruey, Phil Chowienczyk

**Affiliations:** ^1^British Heart Foundation Centre, King’s College London, King’s Health Partners, London, United Kingdom; ^2^Department of Biomedical Engineering, School of Biomedical Engineering and Imaging Science, King’s College London, King’s Health Partners, London, United Kingdom; ^3^World-Class Research Centre, Digital Biodesign and Personalized Healthcare, Sechenov University, Moscow, Russia

**Keywords:** blood pressure measurement/monitoring, central pressure, left ventricular contractility, peripheral pressure

## Abstract

Central systolic blood pressure (cSBP, the peak of the central waveform) is usually regarded as the determinant of peripheral systolic blood pressure with amplification of peripheral systolic blood pressure (pSBP) measured with reference to cSBP. However, the earlier portion of the central waveform up to the first systolic shoulder (P1) may be the major determinant of pSBP. We performed in silico simulation studies and examined previously acquired experimental data (*n* = 131) in which peripheral and central blood pressure waveforms had been acquired both invasively and noninvasively to examine the determinants of pSBP. Measurements were made at baseline and during perturbation of hemodynamics by inotropic and vasoactive drugs. In silico simulations using a central-to-peripheral transfer function demonstrated that pSBP is dependent on P1 and the rate of change (dP/d*t*) of central pressure up to the time of P1 but not cSBP. In computational simulations, peripheral reflection in the radial artery was closely related to dP/d*t*, and 97% of the variability in amplification as measured with reference to P1 was explained by dP/d*t*. In vivo, amplification of pSBP over P1 was correlated with dP/d*t* (*R* > 0.75, *P* < 0.0001 for all data sets), and P1 and dP/d*t* were independently correlated with pSBP, explaining 90% of the variability in pSBP. We conclude that P1 and dP/d*t* are major determinants of pSBP and that pSBP and cSBP are, in part, determined by different cardiac, central, and peripheral vascular properties.

**NEW & NOTEWORTHY** Peripheral systolic BP is determined mainly by the first shoulder and the rate of rise of the central systolic blood pressure waveform rather than the peak of this waveform (central systolic BP). Peripheral and central systolic blood pressure are determined by different cardiac and vascular properties.

## INTRODUCTION

**Figure 1.**
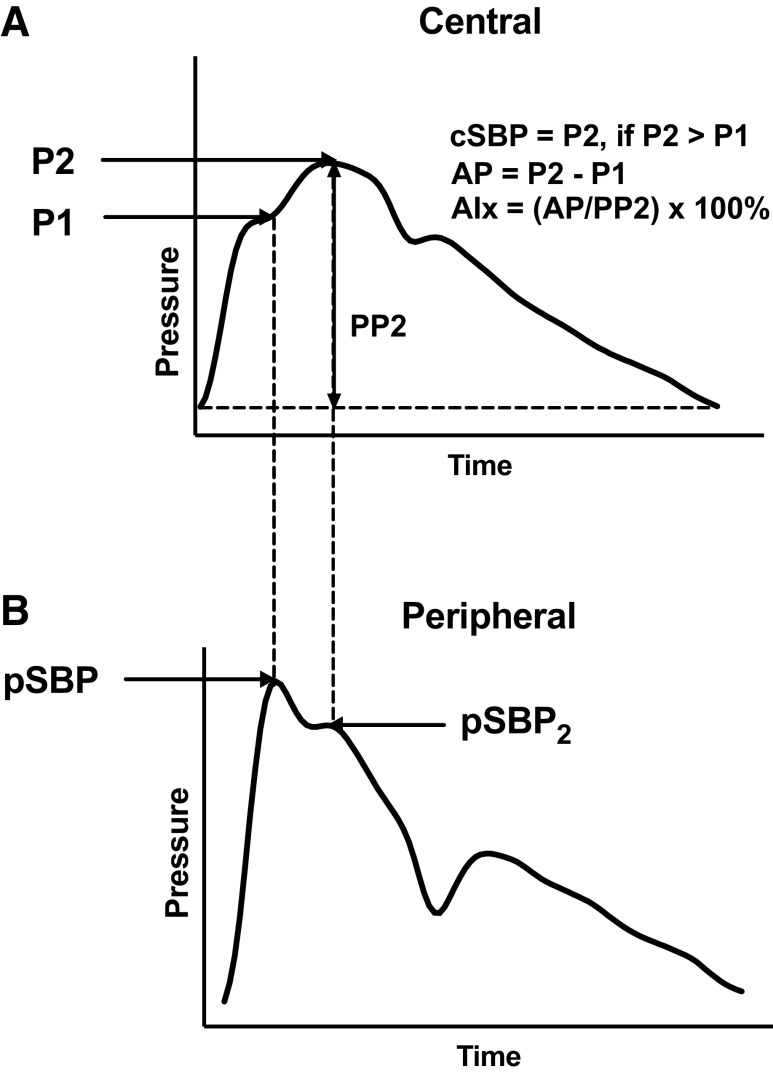
Central (*A*) and corresponding peripheral (*B*) pressure waveforms. AIx, augmentation index; AP, augmentation pressure; cSBP, peak central systolic pressure (usually equal to P2); PP2, pulse pressure at P2; pSBP, pressure at the peak of the peripheral waveform; pSBP2, pressure at the second peak of the peripheral waveform; P1, pressure at the first systolic shoulder of the central waveform; P2, pressure at the peak or second shoulder of the central waveform.

Propagation of the arterial pressure wave from the aortic root toward more peripheral conduit arteries such as the brachial or radial arteries (where blood pressure is traditionally measured) is usually associated with an increase in the amplitude of the pressure pulse wave (1). Much of the literature on this has focused on the relationship between fiducial points on the central and peripheral waveforms, since peripheral systolic blood pressure (pSBP) as the peak of the peripheral waveform is used to assess clinical risk associated with hypertension and to guide acute clinical care. Peak “central” (aortic or carotid) systolic blood pressure (cSBP) usually corresponds to the second systolic peak (P2) of the central pressure waveform (2) (Fig. 1). Peripheral “amplification” of pressure is usually measured as the difference between pSBP and cSBP or as the ratio of peripheral pulse pressure (pPP) to central pulse pressure (cPP) (3). The amplification of pSBP over cSBP is attributed to backward traveling pressure waves reflected from the periphery of the arterial tree wave augmenting a forward going pressure wave in the brachial and radial arteries (4). However, it is likely that the physiological determinant of pSBP is pressure at and preceding the first inflection point of the central systolic waveform (P1) rather than P2 or cSBP (5). Because pressure waves travel from central to peripheral sites at a finite speed (equal to pulse wave velocity), a physical requirement of a component of the central waveform being a determinant of pSBP is that this occurs earlier in the cardiac cycle than pSBP. This is the case for P1, which usually occurs before pSBP, whereas the reverse is true of P2 (P2 occurs after pSBP). Previous studies have shown that cSBP approximates the second systolic peak of the peripheral waveform (pSBP2), which occurs after pSBP (6, 7). This observation is also consistent with the concept of a central wave component that determines a peripheral component arising earlier in the cardiac cycle than the peripheral component.

The purpose of this study was to verify that pressure up to P1 rather than P2 or cSBP is the determinant of pSBP and to examine the determinants, both cardiac and vascular, of the amplification of P1 to pSBP. Theoretical considerations and experimental studies suggest that to a first approximation amplification may be determined by the rate of upstroke of the pressure pulse (dP/d*t*) (8), a measurement related to cardiac contractility (9, 10). dP/d*t*, as defined in this way, represents the magnitude of a pressure wave front and thus may determine the degree of amplification as this wavefront passes from central to peripheral sites. We first examined the relationship of central pressure pulsatile components to the peripheral pulse pressure using a generalized transfer function, which is known to characterize the relation between central and peripheral waveforms over the physiological range of waveform morphology to a close approximation. We performed an in silico modulation of a central waveform, selectively altering P1 and P2, and then used the transfer function to determine which of these affected pSBP. Second, we used a numerical computational model of pulse wave propagation to perform an in silico study of the relationship between pSBP and central waveform features. Finally, we examined pressure waveforms at central and peripheral sites obtained invasively and noninvasively to determine whether theoretical predictions were observed in vivo.

## METHODS

### In Silico Studies: Generalized Central-to-Peripheral Transfer Function

Generalized transfer functions have been widely used to estimate central blood pressures from peripheral blood pressures (11, 12). They involve the application of a mathematical function to the peripheral BP waveform to generate a central waveform, from which cSBP can be obtained as the peak of this waveform. Despite the use of a generalized function that is applied to all subjects irrespective of individual characteristics such as age, gender, and cardiac/vascular properties, such a transfer function has been shown to provide a reasonable transformation of a peripheral to central waveform over the physiological range. This may be because the morphology of the pulse captures the cardiovascular properties that determine the amplification of the central waveform. In this study, we used the inverse of a previously validated transfer function (12) to obtain a central-to-peripheral transfer function. We then modified key features of the central waveform, in particular the amplitude of the points P1 and P2 and the timing (T1) of point P1, to examine the influence of central waveform morphology on peripheral morphology, in particular to determine whether pSBP was more closely related to P2 or P1. In these in silico studies, P1, P2, and dP/d*t* were determined using custom software written in MATLAB (MathWorks, Natick). P2 was identified as the maximum of the waveform and P1 by the zero-crossing point of the second derivative of the pressure waveform with respect to time. In cases in which an inflection point was not seen in the upstroke of the waveform, P1 was taken as the peak of the waveform and P2 as the inflection point in the downslope. dP/d*t* was taken as the ratio of the pulse pressure at T1 (PP1) to T1. P1, P2, and dP/d*t* were modified by scaling values of these points on the pressure waveform and altering the time of P1. Changes in P1 were over the range ±25% (of the mean value) and those in P2 over the range ±25% and were independent of one another. Changes in dP/d*t* were over the range −15% to 60%. Changing P1 did modify dP/d*t* (since the timing of P1 was not altered, a greater P1 resulted in greater dP/d*t*). However, when the time of P1 was altered to modify dP/d*t*, P1 and P2 were unchanged, and so in these cases, change in dP/d*t* was independent of that in P1 and P2.

### In Silico Studies: Computational Model of Pulse Pressure Propagation

We used a previously described 116-segment model (13) that, for a given prescribed aortic flow, can be used to simulate pressure waveforms throughout the arterial tree. Each artery of the network is characterized by its diameter, length, wall thickness, and arterial wall stiffness. The peripheral branches of the model are coupled to three-element Windkessel models that represent the resistive and capacitive properties of the distal microvasculature. For the purpose of this study, a population of 258 virtual subjects with cardiac and arterial parameters spanning the physiological range of the healthy population (13) was used to study the determinants of the peripheral pulse pressure. To examine the contribution of distal pressure wave reflection to amplification, we performed decomposition of the pressure waveform in the radial artery into forward and backward wave components. This was performed using Parker’s time-domain approach (14), based on conservation of mass and momentum, to obtain forward (P_f_) and backward (P_b_) pressure components of radial pulse pressure so that pressure in the radial artery (P_r_) is given by P_r_ = P_f_ + P_b_. P_f_ and P_b_ are given by: $P_{f}=\frac{1}{2}\sum \left(dP+\rho cdU\right)$ and $P_{b}=\frac{1}{2}\sum \left(dP-\rho cdU\right)$. Where *U* is flow velocity, ρ is blood density, and *c* is wave speed, which was calculated using the method of the sum-of-squares (15). The ratio of the magnitudes of P_b_ to P_f_ at the time of pSBP was defined as pulse reflection index (R_PP_). The slope of the upstroke dP/d*t* of the peripheral pressure pulse was defined as the ratio of peripheral pulse pressure (pPP) to time from the start of the pressure upstroke to the time of pSBP_._ dP/d*t* for the central pulse waveform was defined as the ratio of the pulse pressure at P1 (PP1) to T1.

### In Vivo Data

Two previously published data sets in which measurements of central and peripheral pressure waveforms (at the sites summarized in Table 1) were available to investigate the relation between pSBP, cSBP, P1, and the upstroke of the pulse wave. An invasively acquired data set included measurements of central aortic pressure and peripheral (digital artery pressure) acquired during diagnostic angiography in 23 patients (16). Patients with acute coronary syndromes, those with significant valvular disease and rhythm other than sinus rhythm, were excluded from the study. Central aortic pressure was measured using a Millar high-fidelity pressure-tipped catheter (Millar Instruments, Houston, TX) positioned in the proximal aortic root. Peripheral pressure waveforms were acquired simultaneously from the digital artery using a servo-controlled finger pressure cuff (Finometer; Finapres Medical Systems, The Netherlands). We have previously shown that digital artery waveforms obtained in this way are virtually identical to radial artery waveforms acquired by tonometry using the SphygmoCor system (17). Baseline measurements of aortic root pressure and digital pressure were obtained over at least 10 cardiac cycles and ensemble averaged. Sublingual glyceryl trinitrate (GTN, 500 μg) was then administered and further measurements were acquired 2 min after GTN when hemodynamic responses were stable.

**Table 1.** Central and peripheral pressure measurements in in vivo studies

Measurement Sites	Invasive Study	Noninvasive Studies
		Normotensive/Hypertensive Studies
Central site (device)	Proximal aortic root (Millar high-fidelity pressure-tipped catheter)	Carotid artery (applanation tonometry)
Peripheral site (device)	Digital artery (servo-controlled finger pressure cuff)	Radial artery (applanation tonometry)
Signal collected	Pressure waveform simultaneously	Pressure waveform sequentially

The noninvasive data included data from a group of healthy volunteers (*n* = 13 subjects) and hypertensive subjects (*n* = 95) (18, 19). In these data, central blood pressure waveforms were obtained from the carotid artery in which pressure closely approximates aortic pressure (20). Peripheral pressure was measured at the radial artery. In the healthy volunteers, hemodynamic properties were modulated by the administration of drugs with different inotropic and vasopressor/vasodilator properties: dobutamine (DB: 2.5, 5, and 7.5 µg/kg/min), norepinephrine (NA: 12.5, 25, 50 ng/kg/min), phentolamine (PHT: 1 mg bolus + 25 µg/min, 2 mg + 50 µg/min, and 4 mg + 100 µg/min), and nitroglycerin (GTN: 3, 10, 30 µg/min). Radial and carotid pressure waveforms were obtained by applanation tonometry performed by an experienced operator using the SphygmoCor system (AtCor, Australia). Waveforms were obtained at rest in all subjects and during each dose of vasoactive drugs in the normotensive subjects. The sampling rate of the SphygmoCor device is 128 samples per second, and ∼10 cardiac cycles were obtained and ensemble averaged. Brachial blood pressure was measured in triplicate by a validated oscillometric method (Omron 705CP, Omron Health Care, Japan), and the systolic and diastolic blood pressures were used to calibrate radial waveforms. Mean arterial pressure (MAP) was calculated by integration of the radial pressure waveform (sum of data points divided by the number of data points per cardiac cycle). Carotid waveforms were calibrated from MAP and diastolic brachial blood pressures on the assumption of equality of these pressures at central and peripheral sites (21, 22). P1 of all of the measured central pressure waveforms was identified by the in-built software in the SphygmoCor system, which is thought to use the zero-crossing point of the second derivative of the pressure waveform with respect to time (23). In cases in which an inflection point was not identified in the upstroke of the waveform, P1 was taken as the peak of the waveform and P2 as the inflection point in the downslope.

All studies were approved by the Local Research Ethics Committee, and all patients gave written informed consent. Characteristics of subjects are given in Table 2. Full details of the data acquisition process and signal processing for both the invasive and noninvasive data sets have been previously published (12, 19).

**Table 2.** Characteristics of subjects

Characteristics	Invasive Study	Normotensive Controls	Hypertensive Subjects
*n*	23	13	95
Age, yr	62 ± 10	47 ± 10	41 ± 14
Sex, male %	78	77	59
BMI, kg/m^2^	29.1 ± 3.6	–	26.5 ± 9.2
Drug therapy			
ACEI/ARB, %	44	–	51
β-Blocker, %	55	–	16
Calcium channel blocker, %	22	–	39
Diuretic, %	7	–	12
α-Blocker, %	–	–	13
HR, beats/min	61 ± 10	65 ± 8	66 ± 10
pSBP, mmHg	139.9 ± 26.0	116.1 ± 13.7	144.5 ± 21.5
DBP, mmHg	67.0 ± 9.2	69.1 ± 9.9	88.5 ± 13.7
cSBP, mmHg	129.3 ± 23.6	103.8 ± 15.0	134.4 ± 22.1
P1, mmHg	106.5 ± 15.1	96.6 ± 11.0	127.4 ± 17.3
P2, mmHg	129.3 ± 23.6	103.6 ± 15.0	132.5 ± 23.6
dP/d*t*, mmHg/s	493.0 ± 176.3	249.3 ± 63.6	392.9 ± 100.7
LVOT diameter, cm	–	1.87 ± 0.18	1.96 ± 0.25

Values are numbers, percentage, or means ± SD. ACEI, angiotensin-converting enzyme inhibitor; ARB, angiotensin receptor blocker; cSBP, central systolic blood pressure; DBP, diastolic blood pressure; dP/d*t*, rate of upstroke of the pressure pulse; HR, heart rate; LVOT diameter, left ventricular outflow tract diameter; pSBP, peripheral systolic blood pressure; P1, pressure at the first inflection point of the central pressure; P2, pressure at the second inflection point of the central pressure.

### Statistics

Subject characteristics are presented as means ± SD. Multiple regression analysis was used to analyze the relationship between heart rate (HR), time of P1 (T1), and dP/d*t* of central (and peripheral) pressure up to the time of P1 as the potential determinants of peripheral systolic blood pressure and its amplification (pSBP-P1) in the in vivo data. In the hypertensive subjects in whom the sample size was sufficient to allow this, we stratified the analysis by age (<40 or ≥ 40 yr; 40 yr was chosen because it was close to the median age for the group) and by sex, testing for an interaction between these stratifiers, that could influence cardiac and vascular properties, and factors significantly correlated with amplification. Analysis was performed using SPSS v. 22 (SPSS Inc., Chicago, IL), and *P* < 0.05 was taken as significant.

## RESULTS

### Central-to-Peripheral Transfer Function

**Figure 2.**
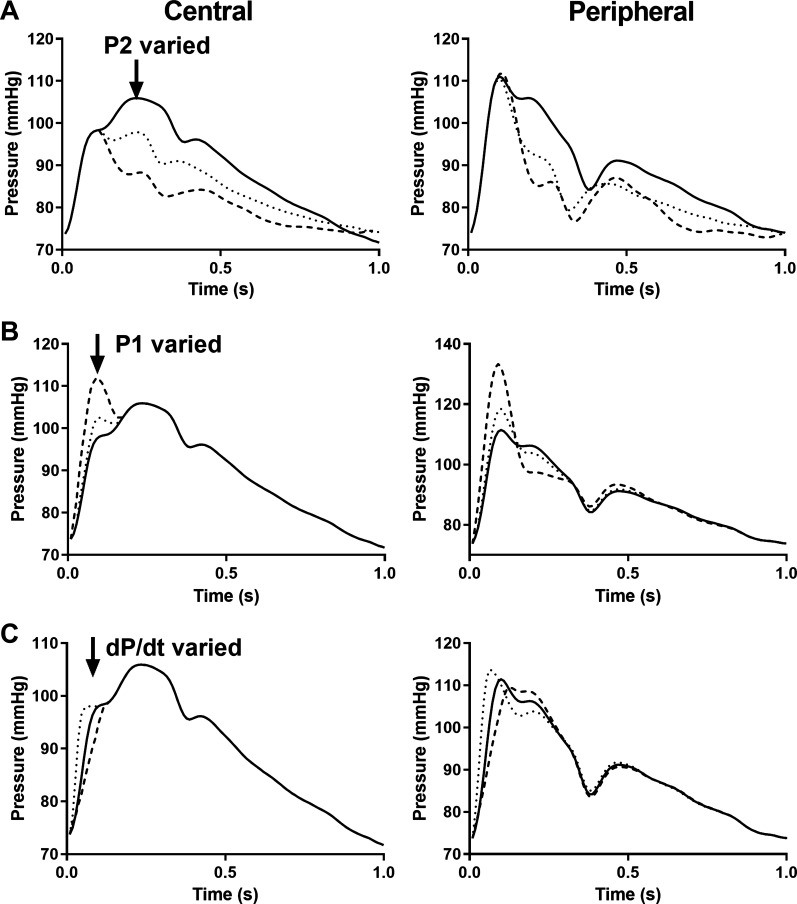
Central pressures (*left*) and the corresponding estimated radial pressures (*right*) derived from the reverse transfer function for different central pressures (solid, dashed, and dotted lines). *A*: modulation of P2 does not influence peripheral systolic blood pressure *B*: modulation of P1 influences peripheral systolic blood pressure. *C*: modulation of dP/d*t* influences peripheral systolic blood pressure. dP/d*t*, change in pressure over time or first derivative of pressure; P1, pressure at the first systolic shoulder of the central waveform; P2, pressure at the peak or second shoulder of the central waveform.

Peripheral pressure waveforms generated by applying the central-to-peripheral transfer function to a typical central pressure waveform are shown in Fig. 2. When the point P2 on the central waveform is modified but P1 is held constant, the value of pSBP remains constant (Fig. 2*A*). Conversely, when P1 is modified and P2 held constant, pSBP changes in proportion to P1 (Fig. 2*B*). pSBP also changes in proportion to dP/d*t* when both P1 and P2 are held constant but the time of P1 is varied (thus altering dP/d*t*, Fig. 2*C*). These results suggest that P1 and dP/d*t* rather than P2 determine pSBP.

### Computational Model of Pulse Pressure Propagation

**Figure 3.**
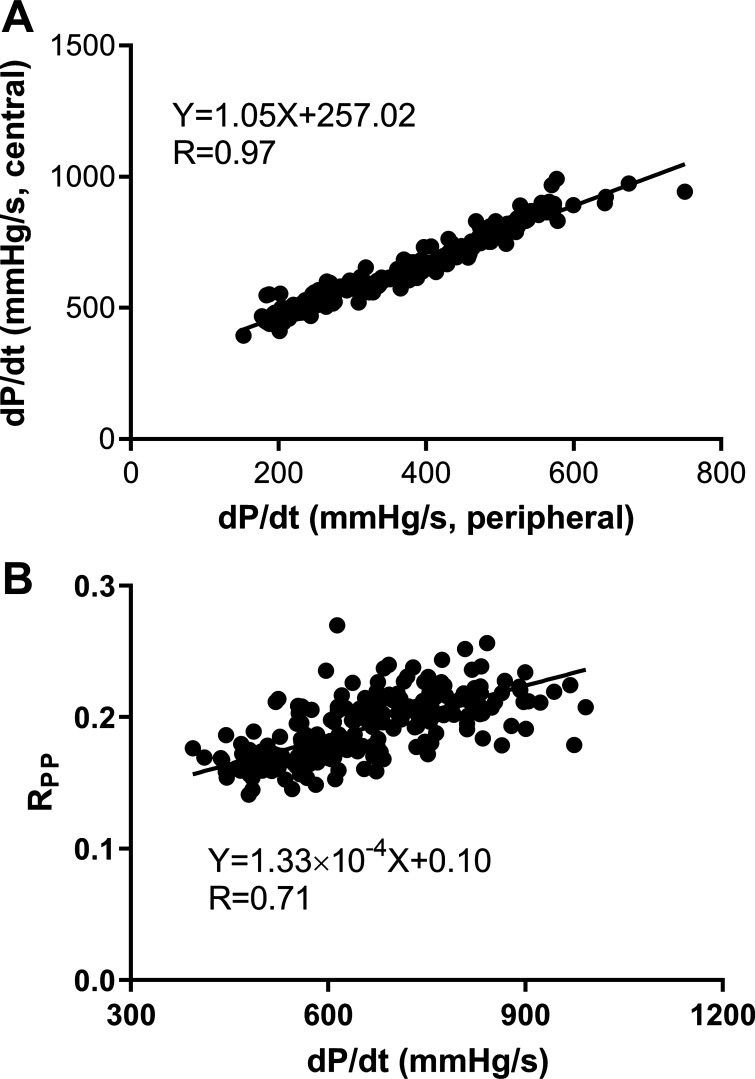
The relationship of dP/d*t* of the peripheral pressure waveform with that of the central pressure waveform and the relationship of the reflection index (R_PP_) with dP/d*t* (peripheral) in the in silico simulation using a computational model of pressure wave propagation. *A*: central vs. peripheral dP/d*t*. *B*: R_PP_ vs. dP/d*t* (peripheral). dP/d*t*, change in pressure over time or first derivative of pressure.

**Figure 4.**
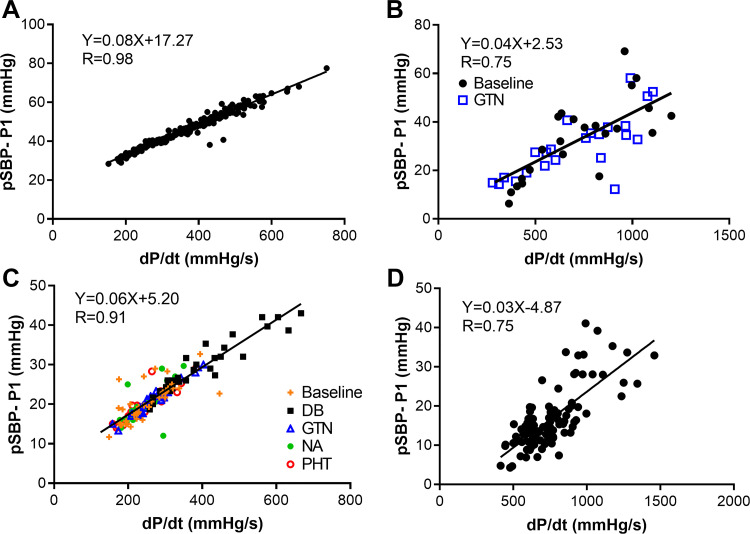
The relationship of peripheral systolic blood pressure (pSBP) amplification above the first shoulder of central pressure (P1), with the rate of rise of central pressure (dP/d*t*). *A*: in silico simulation using computational modeling. *B*: invasive data obtained with aortic pressure wire and Finapres (*n* = 23, 18 male). *C*: normotensive subjects receiving inotropic and vasoactive drugs (*n* = 13, 10 male). *D*: hypertensive subjects (*n* = 95, 56 male). DB, dobutamine; dP/d*t*, change in pressure over time or first derivative of pressure; GTN, nitroglycerin; NA, norepinephrine; P1, pressure at the first systolic shoulder of the central waveform; PHT, phentolamine.

The upstroke dP/d*t* of the radial pressure waveform was highly correlated with that of the central waveform for the virtual population (*R* = 0.97, Fig. 3*A*). Mean wave speed *c* in the radial artery was 8.68 ± 4.27 m/s (means ± SD). The reflection index R_PP_ at the time of peak pulse pressure in the radial artery (Fig. 3*B*) increased with the increase in dP/d*t* (of the central waveform) indicative of a greater backward pressure for any forward pressure with increasing dP/d*t*. For the whole population of virtual subjects, the amplification (pSBP-P1) was mainly determined by dP/d*t* of the central waveform (*R* = 0.98, Fig. 4*A*). A similar result was found for amplification (pSBP-P1) associated with dP/d*t* measured from the peripheral waveform (*R* = 0.95).

### In Vivo Data

**Figure 5.**
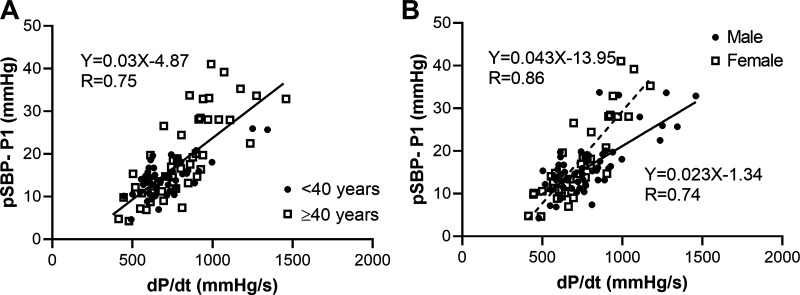
The relationship of peripheral systolic blood pressure (pSBP) amplification above the first shoulder of central pressure (P1), with the rate of rise of central pressure (dP/d*t*) in hypertensive subjects stratified by: age < and ≥40 yr old (47 < 40 yr, 48 ≥ 40 yr old) (*A*) and sex (56 male, 39 female) (*B*). The slope of the relationship between amplification and dP/d*t* was similar in younger and older subjects but was steeper in women than in men. dP/d*t*, change in pressure over time or first derivative of pressure; P1, pressure at the first systolic shoulder of the central waveform.

Amplification (pSBP-P1) was moderately to strongly associated with dP/d*t* measured from the central (or peripheral) waveforms for all the experimental data (*R* = 0.75, 0.91, and 0.75 for the invasive study, normotensive, and hypertensive subjects, respectively, Fig. 4, *B*, *C*, and *D*). The correlation was highest (*R* = 0.91) for the measurements in normotensive subjects, in whom hemodynamics were perturbed and therefore in whom the range of amplification was greatest. When analysis in hypertensive subjects was stratified by age and sex, there was no significant interaction between dP/d*t* and age, but there was a significant interaction with sex, so that the slope of the relation between amplification and dP/d*t* differed in men and women (Fig. 5).

In multiple regression analysis, investigating heart rate (HR), central pressure P1, time of P1 (T1), and dP/d*t* as the potential determinants of pSBP in all experimental data, pSBP was independently correlated to P1 and dP/d*t* (standardized β, 0.707 and 0.252, respectively, each *P* < 0.001) but not with HR or T1. A linear regression model incorporating P1 and dP/d*t* explained 90% of the variability in pSBP. The same analysis performed in the individual study groups gave similar results in each group. When investigating age (in hypertensive subjects) as an additional potential determinant of pSBP, pSBP was independently correlated to P1, dP/d*t*, and age (standardized β, 0.81, 0.172, and 0.092, respectively, each *P* < 0.001 for P1 and dP/d*t*, *P* = 0.005 for age), but age explained <2% of the variability in pSBP. Adjustment for use of antihypertensive drugs did not significantly influence the results.

**Figure 6.**
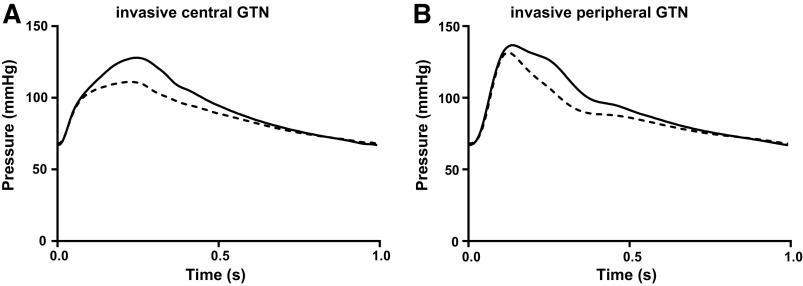
Blood pressure waveform at aortic root measured by an invasive pressure catheter (*A*) and at the digital artery measured by Finapres (*B*) at baseline (solid line) and after nitroglycerin (GTN, dotted line). GTN, glyceryl trinitrate.

In the invasive study, GTN had no significant effect on P1 (106.5 ± 3.2 and 106.1 ± 3.5 mmHg at baseline and after GTN respectively, *P* = 0.84), or on pSBP (139.3 ± 5.4 and 136.4 ± 4.4 mmHg at baseline and after GTN respectively, *P* = 0.16), but reduced cSBP and P2 (P2 = cSBP in all cases in the invasive study) by 16.9 ± 1.8 mmHg (*P* < 0.001). For the pulsatile components of pressure, GTN had no significant effect on PP1 (39.5 ± 2.0 and 38.0 ± 2.9 mmHg at baseline and after GTN respectively, *P* = 0.45), or on pPP (72.9 ± 4.7 and 68.4 ± 3.9 mmHg at baseline and after GTN respectively, *P* = 0.35), but reduced PP2 by 18.0 ± 1.4 mmHg (*P* < 0.001). Average central and peripheral pressures of all subjects at baseline and after GTN are shown in Fig. 6. Thus, as seen in the in silico central-to-peripheral transfer function study, reduction in P2 but not of P1 was without effect on pSBP.

## DISCUSSION

Peripheral systolic blood pressure is the mostly commonly used measure of circulatory function and of cardiovascular risk. The relationship of peripheral pressure with central hemodynamics is of fundamental importance in understanding how peripheral pressure is determined by the interaction of ventricular contraction with the aorta and systemic circulation. The conventional measurement of peripheral pressure amplification as the difference between pSBP and cSBP implies that cSBP is the determinant of pSBP. However, this is implausible since, within each cardiac cycle, pSBP occurs before cSBP and, as previously suggested (5), it is more likely that pressure up to the first shoulder of the central systolic waveform is the determinant of pSBP. In this work, we have confirmed this empirically using a generalized transfer function that encapsulates the relationship between central and peripheral pressure waveform morphology. Importantly, this was by independent manipulation of fiducial points P1, P2, and the timing of P1 (determining dP/d*t*), rather than simply through a correlative analysis.

The phenomenon of amplification can only occur in the presence of pulsatile flow characterized by change in pressure with respect to time, i.e., dP/d*t* generating a wavefront. Using a computational model of the circulation, we showed that reflection of forward going pressure waves in the radial artery (R_PP_) and amplification of pSBP over P1 is closely related to dP/d*t* whether measured centrally or peripherally. Indeed, in the computational model in which, although dependent on assumptions, there is zero experimental error, 97% of the variability in amplification (pSBP-P1) was explained by dP/d*t*. Correlations between amplification and dP/d*t* observed in vivo were more modest but nevertheless strong and persisted despite modulation of cardiovascular properties over a wide range. In regression analysis, dP/d*t* was seen to be the main correlate of amplification. In vivo measurements are inevitably subject to experimental error, both random and systematic, the latter particularly affecting noninvasive measurements that are subject to calibration error. These errors are likely to account, at least in part, for some of the unexplained variation in experimental data. In this regard, the invasive data set we used was probably most robust but still subject to random error, mainly due to movement of the pressure sensor in the aorta. In vivo results using GTN, which changed P2 but not P1 or pSBP, are similar to the in silico modeling. Taken together, the results from our computational modeling and in vivo data strongly support P1 as the major determinant of pSBP and dP/d*t* as the major determinant of amplification of pSBP above P1. However, further work will be required to verify with certainty to what degree dP/d*t* accounts for amplification in vivo and to examine which other factors modify the relationship between amplification and dP/d*t*. In this regard, our analysis suggests that the relationship is in part sex dependent, possibly due to shorter stature of women or other anatomical factors that may increase peripheral reflection (24).

### Perspectives

Our findings have several major implications. If P1 is regarded as the determinant of pSBP rather than cSBP, with amplification as the difference between pSBP and P1, then “true amplification” will show a different relationship to age and other clinical characteristics than the conventional measurement of amplification, as has already been shown in the Framingham study (5) and in our previous computational work (13). Amplification measured as the difference of peripheral and central SBP decreases with age (25), whereas amplification measured as the difference of pSBP and P1 varies little with age (5). The underlying hemodynamic determinants of P1 and P2 differ, with P1 being more closely related to proximal aortic impedance and left ventricular ejection velocities and P2 (usually equal to cSBP) related to the overall compliance of the arterial tree and to ejection volumes (stroke volume to a close approximation) (26, 27). Thus, if P1 is the determinant of pSBP, then pSBP will be determined by different cardiovascular properties to those that determine cSBP and by dP/d*t*. Differential effects of drugs on pSBP and cSBP may be due to their relative effects on cardiovascular properties and on dP/d*t*. This may be a potential mechanism why BP-lowering drugs like β-blockers, which reduce dP/d*t*, may have differential effects on central and peripheral BP (28). The relative risk of pSBP when compared with cSBP may also be related to these underlying hemodynamic determinants rather than the risks of the pressure components per se. The finding that amplification is dependent on dP/d*t* underlines the potential importance of cardiac rather than vascular properties in determining amplification since dP/d*t* of the arterial pressure wave is related to that within the left ventricle and to end-systolic elastance (9, 10). It potentially explains why in very elderly subjects, low peripheral BP and low peripheral amplification may be associated with increased cardiovascular events since low peripheral BP and low amplification could be a surrogate of poor left ventricular function in these subjects (29). Further work will be required to establish to what degree cardiovascular events are related to distinct BP components and dP/d*t*.

In conclusion, peripheral systolic BP is determined mainly by P1, the first systolic shoulder of the central pressure waveform, and the rate of rise of the pressure waveform, which determines the amplification of peripheral SBP above P1.

## GRANTS

This research was supported by the British Heart Foundation (Project Grant PG/17/50/32903). The authors acknowledge financial support from the Wellcome EPSRC Center for Medical Engineering at King’s College London (WT203148/Z/16/Z), Department of Health via the National Institute for Health Research (NIHR) Cardiovascular MedTech Co-operative at Guy’s and St Thomas’ NHS Foundation Trust (GSTT) and the comprehensive Biomedical Research Center and Clinical Research Facilities awards to Guy’s and St Thomas’ NHS Foundation Trust in partnership with King’s College London and King’s College Hospital NHS Foundation Trust, and the Ministry of Science and Higher Education of the Russian Federation within the framework of state support for the creation and development of World-Class Research Centers Digital Biodesign and personalized healthcare (075-15-2020-926).

## DISCLOSURES

No conflicts of interest, financial or otherwise, are declared by the authors.

## AUTHOR CONTRIBUTIONS

Y.L., A.G., P.H.C., S.V., J.A., and P.C. conceived and designed research; A.G. performed experiments; Y.L. analyzed data; Y.L., P.H.C., S.V., J.A., and P.C. interpreted results of experiments; Y.L. prepared figures; Y.L. drafted manuscript; Y.L., A.G., P.H.C., S.V., J.A., and P.C. edited and revised manuscript; Y.L., A.G., P.H.C., S.V., J.A., and P.C. approved final version of manuscript.
